# Content of Capsaicinoids and Capsiate in “Filius” Pepper Varieties as Affected by Ripening

**DOI:** 10.3390/plants9091222

**Published:** 2020-09-17

**Authors:** Mercedes Vázquez-Espinosa, Oreto Fayos, Ana V. González-de-Peredo, Estrella Espada-Bellido, Marta Ferreiro-González, Miguel Palma, Ana Garcés-Claver, Gerardo F. Barbero

**Affiliations:** 1 Department of Analytical Chemistry, Faculty of Sciences, University of Cadiz, Agrifood Campus of International Excellence (ceiA3), IVAGRO, Puerto Real, 11510 Cadiz, Spain; mercedes.vazquez@uca.es (M.V.-E.); ana.velascogope@uca.es (A.V.G.-d.-P.); estrella.espada@uca.es (E.E.-B.); marta.ferreiro@uca.es (M.F.-G.); miguel.palma@uca.es (M.P.); 2 Centro de Investigación y Tecnología Agroalimentaria de Aragón, Instituto Agroalimentario de Aragón-IA2, CITA-Universidad de Zaragoza, 50059 Zaragoza, Spain; ofayos@cita-aragon.es (O.F.); agarces@cita-aragon.es (A.G.-C.)

**Keywords:** capsaicinoids, capsiate, *Capsicum* spp., Filius variety, fruit ripening, UHPLC

## Abstract

Peppers are fruits with wide genetic variability and multiple ways of being consumed that hold a relevant position in the human diet. Nowadays, consumers are interested in new gastronomic experiences provided by pepper cultivars that present new shapes, colors, and flavors while preserving their bioactive compounds, such as their capsaicinoids and capsinoids. However, numerous changes take place during their development that may alter their biological properties. Therefore, this work evaluates the capsaicinoid and capsiate contents in two traditional varieties of ornamental peppers (“Filius Blue” and “Filius Green’”) during fruit maturation. The aim is to determine the ideal harvesting moment depending on the farmer’s objective (e.g., achieving a specific color, shape, or flavor; achieving the maximum concentrations of bioactive compounds). The capsaicinoid contents followed different patterns in the two varieties analyzed. The “Filius Blue” variety exhibited increasing concentrations of capsaicinoids up to the 41st day post-anthesis (dpa), from which point on this trend was reversed. The concentrations in the “Filius Green” variety increased and decreased several times, reaching maximum concentrations on the 69th dpa. Regarding capsiate contents, both varieties varied in the same way, reaching maximum concentrations on the 34th dpa and then decreasing.

## 1. Introduction

Peppers belong to the Solanaceae family from the genus *Capsicum*, which is native to Central and South America. They are cultivated in tropical and warm climate regions worldwide [1]. Peppers are fruits that hold a significant position in human diets because of their versatility, which allows them to be consumed fresh in salads, fried, boiled, in their dehydrated form as a seasoning, or even as a sauce or jam [2]. Peppers have ample genetic diversity and comprise a substantial number of varieties that differ in plant size (from short, compact plants to plants as tall as three to four feet), color (green, purple, yellow, chocolate, orange, or red, depending on the pepper variety and maturation stage), flavor (from the non-pungent varieties to the hottest species), shape (round, elongated, wide, narrow, as well as special shapes such as bells), and pepper size (from small to full-size fruits) [3]. This diversity is the reason for the remarkable potential for peppers to be used in the agri-food industry, either as coloring or flavoring agents or in forms that utilize their sensory characteristics [4].

Sometimes, when we eat a food, we seek a certain sensation; however, a colorful dish can also be more appetizing. For this reason, in recent years new consumption trends have increased considerably, since current consumers seek new culinary experiences, resulting in the demand for novel cultivars with new morphologies, colors, textures, flavors, and fragrances. In this sense, there has been an increase in the commercialization and consumption of new hot pepper varieties that meet all of these premises, which have gained consumers’ interest [5]. One of these new pepper varieties, known as “Filius” (*Capsicum annuum* L.), stands out amongst the rest, since it satisfies this new consumption trend and therefore has considerable marketing potential. This variety is characterized by its small, ovate shape and its numerous spicy fruits that grow vertically on the plant. The “Filius” variety can be found in blue and green cultivar forms, according to the color of their immature fruit, which are blue-purple and green, respectively. Both cultivars of “Filius” peppers mature to a red color [6].

Additionally, consumers appreciate the valuable health properties that are attributed to this food group. In fact, peppers are known to have also been used as medicinal plants because of their substantial contents of bioactive compounds, which provide health benefits to consumers [7,8]. Capsaicinoids and capsinoids, which are among those bioactive compounds of interest, are also responsible for the pungency of chili peppers, which is one of their most important commercial traits [9,10]. Therefore, both capsaicinoids and capsinoids have received great attention from consumers because of their extensive pharmacological and physiological effects, i.e., antitumor [11], antioxidant [12], antiobesity [13], anti-inflammatory [14], and analgesic [15] effects. The basic chemical structure of capsaicinoids is formed by a combination of vanillylamine and a branched-chain fatty acid via an amide moiety; capsaicin and dihydrocapsaicin are the most significant capsaicinoids. Capsinoids have a similar structure except for their central bond, which is a combination of vanillyl alcohol with a branched-chain fatty acid via an ester group [16,17]. Capsinoids were first isolated from a low-pungengy variety known as *C. annuum* cv. CH-19 Sweet, with capsiate and dihydrocapsiate being the major capsinoids in nature [18]. Although their main difference lies with the fact that they present either amide or ester bonds, capsinoids are not spicy; that is, they do not produce an intense burning sensation. The pungency of capsinoids has been estimated at approximately 1/1000 compared to that of capsaicinoids. This makes them more propitious and appealing for consumption, which in turn supports their inclusion in both food and dietary supplements, as well as in other products with medicinal purposes [19]. The receptor vanilloid type-1 (TRPV1) is the one responsible for the burning sensation. Due to their slightly different structure, capsinoids do not stimulate the pungency receptors in the mouth; however, their effect in the intestines results in similar physiological activities [20].

The biosynthesis of capsaicinoids and capsinoids begins in the placenta in peppers, although they can then be excreted and detected in the seeds or pericarp [21]. The presence of capsaicinoids and capsinoids in peppers is controlled by the single dominant gene *Pun1* [22]. However, the accumulation of one of the two compound types over the other seems to be due to the action of the putative aminotransferase (*pAMT*) gene, which encodes an aminotransferase (pAMT) involved in the production of vanillylamine from vanillin. The mutations that have been described in the *pAMT* gene result in a loss-of-function of *pAMT*, which in turn increases the production of vanillyl alcohol over that of vanillylamine, and consequently capsinoid accumulation dominates over capsaicinoid production [23].

While the genotype clearly plays a significant role with regards to the content and diversity of these bioactive compounds in peppers, the environmental conditions and agronomic practices can interact with their genotypes and have an influence on the bioactive properties of the final food [24]. It seems clear that numerous changes—such as the degradation and synthesis of fruit metabolites—may take place over the different stages of the ripening process of pepper fruits [25]. The characterization of the phytochemical changes that take place over the peppers’ ripening process is particularly interesting from dietary and nutritional perspectives, since these changes may affect antioxidant activities, aroma, taste, quality, and ultimately consumers’ preferences. Peppers are harvested and consumed at different ripening stages, from immature to fully ripe. It is, therefore, very important to be able to determine their optimal harvesting time during the ripening process. For this purpose, it should be kept in mind that the optimum color stage for a culinary application may not coincide with the moment of maximum bioactive compounds concentration, since each characteristic follows different development patterns [26]. Therefore, and depending on the attributes that are sought by producers (color, pungency, etc.), the fruit optimal harvesting moment will vary [7,27].

Nowadays, peppers are mainly used for cooking; however, when they were first brought over to Europe in the 15th century, they were highly appreciated as ornamental plants, rather than food. Ornamental plants are those plants that are grown for their beauty and generally used in gardening, interior decoration, and landscaping [28]. Therefore, they should exhibit some remarkable morphological characteristics that support their aesthetic value, such as small, erect, and colorful fruits that present a color contrast with the foliage over the whole ripening process. Other positive features would include: vivid foliage, easiness to grow, long durability and the possibility of being grown in small pots [29,30,31]. The subsequent search for new shapes and colors as well as new flavors when used as food encouraged their cultivation and commercialization. In fact, the interest in these new pepper varieties has increased worldwide to eventually become a considerable financial source for growers [32].

The changes in both individual and total capsaicinoids concentrations, as well as capsiate contents, over the fruit ripening period, has already been analyzed in several pepper varieties, including some super-hot, pungent, and non-pungent varieties [33,34,35]. However, given the great variability between the different varieties of peppers and the growing conditions, it is necessary to deepen this type of study to have more precise knowledge about the evolution of these beneficial compounds throughout maturation. Moreover, other traditionally ornamental varieties, such as the one studied here, which have been recently incorporated to our diets despite their smaller fruits, have not yet been analyzed for capsaisinoids and capsiate contents. Only the Peter pepper variety has been analyzed just for capsaicinoids content [36]. These types of ornamental varieties can be used for food and dishes with a dual purpose (nutritional and decorative) due to the large amount of nutritious and healthy compounds and for aesthetic or decoration purposes, thanks to the variety of colors that they present during the ripening stages. Therefore, this work intends to apply Ultrasound-Assisted Extraction (UAE)—an extraction method that is well known to be efficient and easy to use [37]—plus Ultra-High-Performance Liquid Chromatography (UHPLC) to determine capsaicinoids and capsiate accumulation in two ‘Filius’ pepper cultivars over their ripening process. Additionally, fruit color changes over the ripening process have been registered, so that they can be used as a guidance to select their optimal harvesting moment depending on their final purpose.

## 2. Results and Discussion

### 2.1. Changes in Peppers’ Total Capsaicinoids Content

**Figure 1:**
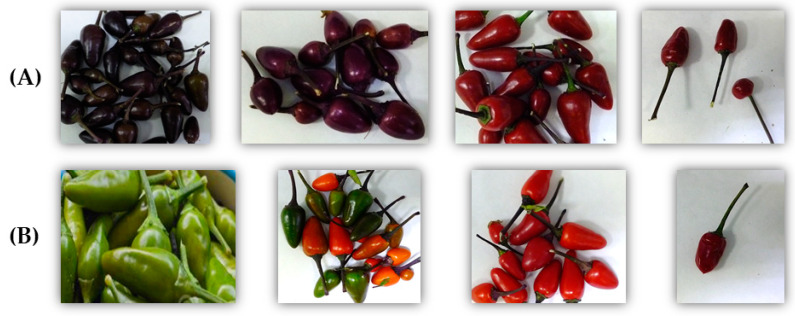
Changes in the color of the fruits in both varieties over their maturation process. (**A**) Purple variety ‘Filius Blue’; (**B**) green variety ‘Filius Green’. The images of each variety were taken at 13, 34, 55 and 76 dpa from left-to-right, respectively. The respective UHPLC-PDA chromatograms (λ = 280 nm) are included in Figure S1 of Supplementary Material.

The two cultivars under study used to perform the experiments were grown in an automated and acclimatized greenhouse under controlled conditions. They began to produce peppers during the second week of July and they were harvested 11 weeks later, specifically on 30 September. They were monitored during the development of the pepper fruits, from the 13th until the 76th day post anthesis (dpa). The visual appearance of the peppers at the different development stages was registered and is presented in Table 1 and Figure 1.

**Figure 2:**
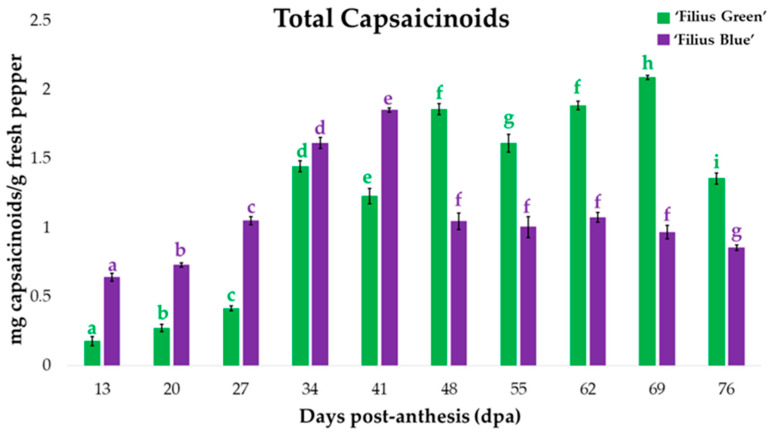
Total capsaicinoid concentration (mg g^−1^ fresh weight (FW)) during fruit ripening (*n* = 2). The same letter assigned to each variety indicates that there was no significant variation in capsaicinoids content according to Tuckey’s test, that is, both values have a *p*-value > 0.05. Letter colors correspond to their respective variety, i.e., green for ‘Filius Green’ and purple for ‘Filius Blue’.

Once the peppers of both varieties were obtained in each of the maturation stages, the seeds and stems were discarded. The pericarp and placenta were ground together, forming the sample for analysis. These biological samples were totally homogenous and representative. A quantity between 232–346 g of peppers for each dpa were collected from 10 pepper plants of each genotype, cultivated in a greenhouse, to avoid any particular effect from individual pepper fruits [38]. Two technical replications were performed in each maturation stage; thus, the figures throughout the manuscript show the mean of both results. As can be seen in Figure 2, total capsaicinoids concentration varied significantly depending both on the particular cultivar and the development stage of its fruits. A substantial variation in capsaicinoids content could be attributed to the genotype, as well as to environmental factors and growing conditions [39]. However, it should be remembered that, in this case, the environmental and growing conditions were controlled, being the same for both varieties. Moreover, Olguín-Rojas et al. and Vázquez-Espinosa et al. grew their pepper cultivars under similar conditions to those in the present study and did not report the same trends for the total amount of capsaicinoids [34,40]. This may suggest that the changes in the contents could be attributed to each pepper genotype. In both varieties analyzed, capsaicinoids were not detected until the 13th dpa.

Most studies on the accumulation of capsaicinoids in *Capsicum* fruits have shown an increase in the concentration of these compounds in the early stages of the fruit development. This climbing trend remains during part of the ripening process until a maximum value is reached, approximately on the 40th dpa. Finally, a gradual degradation of these compounds is observed that ranges from 30% to 65% depending on the different pepper cultivars [41,42,43,44]. The purple variety ‘Filius Blue’ analyzed in this work shows exactly that trend. The concentration of total capsaicinoids increases from the first days of maturation until a maximum value is reached on the 41st dpa (corresponding to 1.85 mg g^−1^ concentration). After that day, the trend is inverted and a marked 44% reduction in capsaicinoids content on the 48th dpa was registered. This change may be associated both with a considerable inhibition in the biosynthesis of these compounds [45], and with the action of peroxidases, which are enzymes capable of degrading capsaicin (C) and dihydrocapsaicin (DHC), as evidenced by the results of in vitro experiments performed by Bernal et al. [46,47]. Capsaicin is synthesized in the placenta and accumulates in the vacuoles of placental epidermal cells until it is metabolized [48]. The activity of basic peroxidases may be directly related to this catabolic reaction. The arguments that support a relevant role by peroxidase in the degradation of capsaicin are based on the unique location of peroxidase in the placental epidermal cells of hot pepper fruits. Furthermore, this evidence is supported by the strong oxidizing activity of capsaicin on basic peroxidase isoenzyme [49,50]. Finally, during the last stages of fruit ripening, the total capsaicinoid content remains practically constant until the end of their maturation.

Unlike what is generally reported in the literature, the green variety ‘Filius Green’ presented a significant increase in the content of capsaicinoids at the early stages of the peppers’ development (between the 13th and the 34th dpa, when it reached 1.44 mg g^−1^), followed by two more moderate increments around the end of the ripening process. In addition, two perceptible decreases were observed in the amount of capsaicinoids during the maturation period: a decrease of approximately 15% was observed between the 34th and the 41st dpa, and the second one by 13.5% was registered between the 48th and 55th dpa. Finally, over the last stages of maturation, a considerably greater drop of around 35% was observed in the total content of capsaicinoids. These decreases in capsaicinoids content could be due to both the action of the peroxidases and the reduced synthesis of capsaicinoids in the peppers due to the specific cultivation conditions in the greenhouse [49]. Comparable capsaicinoid accumulation patterns, with maximum capsaicinoid content during the last developmental stage, have been previously described for a number of cultivars such as Habanero, which exhibited maximum content levels on the 63th dpa [51], Habanero Roxo, with maximum levels on the 62nd dpa [34], and Malagueta on the 68th dpa [33]. It is believed that this behavior could be attributed to the particular growing conditions in the greenhouse, where temperature, humidity, irrigation, and fertilization were all controlled [24].

As mentioned above, nowadays, consumers look for new gastronomic experiences, including different colors of pepper fruits, but the desired color may not coincide with their maximum concentration of bioactive compounds.

If consumers are looking for less spicy sensations, they should collect the peppers during the early stages of the fruit development, when they present lower capsaicinoids concentrations and the varieties studied exhibit purple and green colors. After 1 month of maturation (around 27–34 dpa), the fruits maintain a purple or green color, respectively; however, their capsaicinoids content is considerably larger, so that consumers can experience sensations of greater pungency, despite the fact that their visual appearance remains unaltered. On the other hand, capsaicinoids content in the ‘Filius Blue’ variety is very similar on the 27th dpa, and between the 48th and the 69th dpa, which means that consumers can experience similar flavor sensations despite their different appearance, since in the first case the fruits present a green color, while during the second period they exhibit a red color. Finally, over the last stages of fruit development, that is, when they look red, their capsaicinoids content is relatively high in all cases, so that their consumption is preferably associated to the seeking of pungent sensations.

### 2.2. Changes in the Content of Individual Capsaicinoids

**Figure 3:**
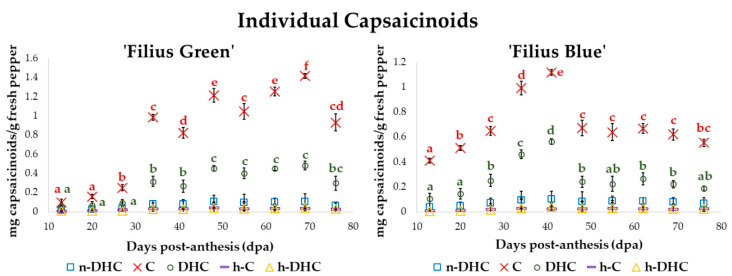
Individual capsaicinoids concentrations (mg g^−1^ fresh pepper (FW)) over the fruit ripening process (*n* = 2). The same letter indicates that no significant difference according to Tuckey’s test were observed, that is, both values have a *p*-value > 0.05. In addition, a different letter color has been assigned to each specific capsaicinoid, i.e., blue for nordihydrocapsaicin (n-DHC), red for capsaicin (C), green for dihydrocapsaicin (DHC), purple for homocapsaicin (h-C), and yellow for homodihydrocapsaicin (h-DHC). Significant differences in the two major compounds have been highlighted to improve readability.

Five capsaicinoids—nordihydrocapsaicin (n-DHC), capsaicin (C), dihydrocapsaicin (DHC), homocapsaicin (h-C), and homodihydrocapsaicin (h-DHC)—were identified in the two varieties of pepper. The content of each individual capsaicinoid in the pepper samples during the ripening of the fruit is displayed in Figure 3. The analyses were carried out in duplicate for each developmental stage (dpa).

**Figure 4:**
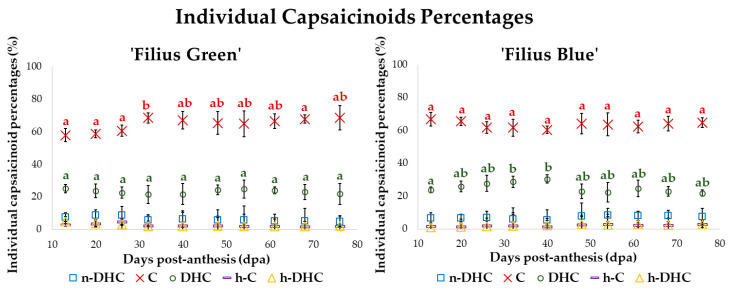
Individual capsaicinoid percentages (%) during the fruit ripening (*n* = 2). The same letter has been assigned in this figure to indicate that there are no significant differences according to Tuckey’s test, that is, both values have a *p*-value > 0.05. In addition, a different letter color has been assigned to each specific capsaicinoid, i.e., blue for nordihydrocapsaicin (n-DHC), red for capsaicin (C), green for dihydrocapsaicin (DHC), purple for homocapsaicin (h-C), and yellow for homodihydrocapsaicin (h-DHC). Significant differences in the two major capsaicinoids have been highlighted to improve readability.

C was the one to present the highest content over the entire maturation period of the fruit in both varieties, followed by DHC, and then a small amount of n-DHC. Other capsaicinoids, such as, for instance, h-C and h-DHC, were detected in minimal amounts. The capsaicinoids content profile that was obtained is consistent with the results reported by similar studies [52,53]. C and DHC are considered the major capsaicinoids to contribute to the typical pungency of chili peppers that causes a heat sensation in the mouth, palate, throat and back tongue [54]. It was also observed that the changes registered for each individual capsaicinoid throughout the development of the fruits followed a similar pattern to that of the total capsaicinoids previously explained. The percentages of each specific capsaicinoid were also calculated during the maturation of the fruit and are represented in Figure 4.

In both varieties, it was observed that the percentage of each individual capsaicinoid remained practically constant during the maturation period, with just a slight variation. C content rated around 60–70% during fruit ripening. Similarly, DHC changed between 20–30%; and both together represent approximately 90% of the total capsaicinoids content. These results were similar to those reported in the literature for other varieties [53,55]. However, they differed from the description by Barbero et al. for the Peter pepper variety, where the percentage of C and DHC reversed during the maturation of the fruit. In the study of Barbero et al. DHC was the major capsaicinoid over the early stages and C over the final period [36]. In other pepper varieties, DHC was registered as the main capsaicinoid [56]. It can also be noticed that a decrease in the percentage of C content corresponds to an increment in the percentage of DHC and vice versa. n-DHC was the third major capsaicinoid at between 5% and 8% of the total capsaicinoid content depending on the fruits ripening stage. Lower percentages of around 1–3% were registered for the minor capsaicinoids, h-C and h-DHC.

### 2.3. Changes in the Standardized Value of Capsaicinoids

The standardized values of the main capsaicinoids were determined according to the following equation:

$$RP_{i}=\frac{Cx_{i}}{CT_{i}}\times 100$$

where *RPi* is the relative percentage of each capsaicinoid, *C_i_* is the concentration of the selected capsaicinoid at “*x*_i_” dpa, and *CT_i_* is the maximum concentration of that particular capsaicinoid during the whole ripening process.

It can be seen from Table 2 that, in both varieties analyzed, the content of most individual capsaicinoids, with the exception of h-C, followed a similar pattern. In addition, they all follow the same trend as for total capsaicinoids content previously explained for each one of the two varieties under study.

With regards to the ‘Filius Green’ variety, all the capsaicinoids showed a fairly similar behavior and reached their maximum content level of n-DHC, C, DHC, and h-DHC on the 69th dpa. On the other hand, h-C reached it at an earlier stage of maturation, specifically on the 48th dpa, at which point the rest of the capsaicinoids were also very close to their maximum content level.

Regarding the ‘Filius Blue’ variety, the relative percentages of n-DHC, C, DHC, and h-DHC increased until the 41th dpa, when they reached their maximum concentration during the fruit development process. From that moment on, there was a gradual decrease in their concentration, then there is a slight increase on day 62th dpa, to finally decrease again until the end of the fruit maturation. Contrarily, h-C reached its maximum concentration some days later, so that its relative percentage increased until the 34th dpa, when it reached levels close to its maximum concentration. Then, it went down slightly and increased again to reach its maximum value on the 55th dpa. A gradual concentration drop took place over the last stages and finally a new increment that could be attributed to the effect of fruit dehydration. Most of the pepper varieties described in the literature present a standardized evolution pattern that is specific for each one of the main capsaicinoids present in their fruits. In fact, not all the particular capsaicinoids that can be found in a particular variety exhibit the same trend for their normalized values and, therefore, each particular capsaicinoid reach its maximum concentration at a specific moment during the maturation process [33,36,41]. However, Vázquez-Espinosa et al. reported that in the ‘Naga Jolokia’ pepper variety, all the capsaicinoids followed the same pattern throughout the ripening process [40].

In this work, the variation of bioactive compounds in each variety are mainly due to their genotype, since they have been grown under the same controlled conditions. However, the same variety may be cultivated under different environmental conditions, which would also affect its compounds content. In those cases, cultivation parameters should be closely controlled so that the resulting measurements are reproducible and comparable to those obtained by other researchers.

### 2.4. Changes in Capsiate Content during Fruit Ripening

First of all, it should be noted that capsinoids, in both varieties, were detected in smaller amounts than capsaicinoids throughout the whole fruit ripening period. Specifically, capsiate (CTE) content represented around 5–30% with respect to the total amount of capsaicinoids, depending on the developmental stage.

**Figure 5:**
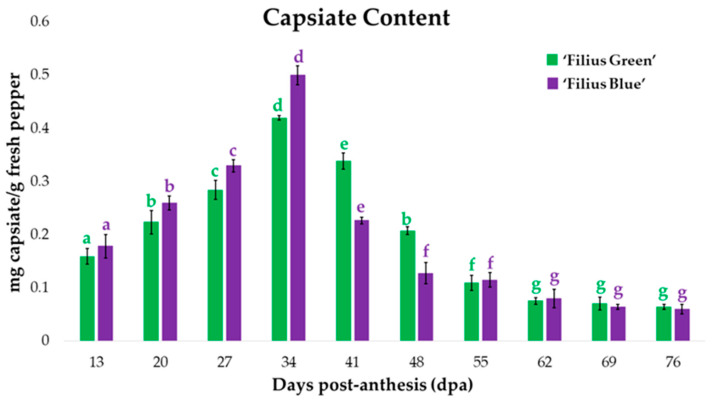
Capsiate concentration (mg g^−1^ FW) during fruit ripening (*n* = 2). The same letter assigned to each variety in this figure indicates that there were no significant differences in capsaicinoids content according to Tuckey’s test, that is, both values have a *p*-value > 0.05. In addition, a different letter color has been assigned to each specific variety, i.e., green for ‘Filius Green’ and purple for ‘Filius Blue’.

Regarding the CTE accumulation pattern over the peppers development (Figure 5), a similar trend was observed for the two pepper cultivars. The CTE content increased until the 34th dpa, when it reached its maximum value (0.42 mg g^−1^ of FW for ’Filius Green’ and 0.50 mg g^−1^ of FW for ‘Filius Blue’), and, from then on, its concentration decreased drastically until the 62nd dpa by 82% and 84%, respectively. Such a drastic reduction in capsiate content after reaching its maximum value may be related either to a decrease in the expression of the biosynthetic structural genes of capsinoids [57] or to the action of the peroxidase enzymes present in peppers, similar to what has been previously explained for capsaicinoids [47]. Capsinoids also accumulate in cell walls and vacuoles [48]. In this sense, Lema et al. suggested that the same peroxidases that oxidize capsaicinoids are also capable of oxidizing capsinoids [58]. Finally, capsinoids content remained constant over the last stages until the end of the fruit maturation process.

These results are in agreement with those described for other *C. annuum* varieties with different pungency levels. According to Yazawa et al., CTE and dihydrocapsiate (DHCTE) reached their maximum content on the 30th dpa and then decreased [59]; Jang et al. also registered the highest capsinoid content between the 30th and the 40th day of the fruit ripening period [60]; Fayos et al. reported that capsinoids content in the varieties ‘Chiltepín’, ‘Tampiqueño 74′ and ‘Bhut Jolokia’ increased to its maximum value on the 40th dpa [35].

Another fact to keep in mind is that the CTE accumulation pattern in the green variety ‘Filius Green’ was different from that of capsaicinoids’. Whereas CTE content increased up to the 34th dpa and then decreased to a constant level, capsaicinoids content had a series of ups and downs throughout the whole maturation process of the fruit and reached its maximum value on the 69th dpa. These differences in trends could be attributed to the presence of a number of factors that would act as regulators of the accumulation of capsinoids and capsaicinoids in the biosynthesis pathway of this cultivar [61]. However, with regards to the purple variety ‘Filius Blue’ the accumulation pattern for both capsaicinoids and CTE was the same, even if the maximum value of capsiate content was reached at an earlier stage of maturation. This could be due to the greater instability exhibited by capsinoids either in water or under high temperatures with respect to that of capsaicinoids’. Other methodologies were not tested because capsinoid extraction has been previously optimized in our research group using Ultrasound-Assisted Extraction (UAE) and Microwave-Assisted Extraction (MAE) [62]. Our previous work demonstrated that quantitative capsinoid extractions were achieved using these methods. Since no significant differences were observed between both techniques, UAE was used for availability. Natural capsinoids are probably stable in non-polar solvents and labile in polar solvents and they probably tend to decompose in protic solvents such as alcohol or water [63]. Due to the nutraceutical contributions attributed to capsiate, that is, because of its attractive beneficial properties for human health, pepper fruits should be consumed when they present the largest concentration of this compound. According to the data resulting from this study, that moment would take place during the first stages of the fruit development, specifically between the 20th and 41st dpa for both varieties. During that period of time, the fruits exhibit green and purple hues, respectively.

## 3. Materials and Methods

### 3.1. Reagents

The methanol (99.9%) and acetonitrile (99.9%), of HPLC grade used for the extraction and chromatographic separation, respectively, were purchased from Panreac Química S.L.U. (Castellar del Vallés, Barcelona, Spain). The glacial acetic acid (99%), also of HPLC grade, was obtained from Merck (Darmstadt, Germany). The ultra-pure water was supplied by a Milli-Q water purification system manufactured by EMD Millipore Corporation (Bedford, MA, USA). The capsaicinoids reference standards, i.e., capsaicin (97%) and dihydrocapsaicin (90%) were purchased from Sigma-Aldrich Chemical Co. (St. Louis, MO, USA). The capsiate standard was synthesized following the method described by Barbero et al. [64] from the Department of Organic Chemistry at the University of Cadiz.

### 3.2. Pepper Cultivar and Growing Conditions

Ten plants of each genotype were grown in an automated greenhouse, under controlled conditions, at the Agrifood Research Centre of Aragón (CITA-Zaragoza, Zaragoza, Spain). The experiment was carried out over the spring-summer season (April–September 2016) with an average day/night temperature of 24/14 °C during the spring and 27/19 °C during the summer. The plants were distributed randomly within the greenhouse in 17 cm diameter and 18 cm height black plastic pots (one plant per pot). The pots were filled with a substrate mixture formed by peat, sand, and clay-loam soil as well as Humin Substrat (Klasman-Deilmann, Geeste, Germany) (1:1:1:1 *v*/*v*/*v*/*v*). They were enriched with two grams of a slow-release fertilizer (Oscomote 16N-4P-9K, Scotts, Tarragona, Spain), and watered by means of a drip irrigation system to maintain their optimum humidity levels for growth.

The plants began to bloom by mid-July and the flowers were marked, at the onset of their anthesis, in 7-day intervals. The pepper fruits were harvested during the last week of September at different ripening stages, from immature to senescent. The fruits from the ‘Filius’ variety were harvested and grouped according to ten different stages of development: 13, 20, 27, 34, 41, 48, 55, 62, 69, and 76 days post-anthesis (dpa), and between 232 and 346 g were collected for each pepper sample. The pepper seeds and stems were discarded prior to analysis. The pericarp and placenta were ground together to a fine pulp by means of an Ultra-Turrax blender until a fully homogenous mass was obtain. All the samples were stored at −20 °C until analysis.

### 3.3. Ultrasound-Assisted Extraction of Fresh Pepper

The ultrasound equipment was mainly formed by three components: an ultrasound Sonoplus probe (BANDELIN ELECTRONIC, Heinrichstraβe, Berlín, Germany), a thermostatic bath with a 7 L refrigerated circulator (PolyScience, Niles, IL, USA), and a double-walled vessel (the double mantle allowed us to control the temperature of the medium by means of additional cooling/heating systems).

The methanolic pepper extracts at the different maturation stages were prepared according to the methodology that had been previously developed by our team for capsaicinoids [65] and capsinoids [62]. The pepper pulp from each one of the ripening stages was weighed into a plastic tube, which were filled up to the desired volume with the corresponding solvent. Then, the plastic tubes were placed in the ultrasound bath to start the extraction process. Ultrasonic power, extraction temperature and sonication time were controlled via the equipment panel. All the samples were extracted in duplicate, and then the extracts were centrifuged twice for 5 min at 7500 rpm (9.5 cm orbital radius). The supernatant from either of the centrifuged was collected into the same 25 mL volumetric flask, which were then made up to the mark with the same solvent. Finally, the extracts were maintained at −20 °C until analysis.

The extraction methods used to carry out the analyses [62,65] had been previously developed and optimized in our research group and present a repeatability and intermediate precision lower than 5%. Furthermore, both extraction methods are quantitative. For these reasons, we considered that it was not necessary to perform a greater number of technical replications since the methods have been proven to be efficient, accurate and adequate, and the samples employed were totally representative.

### 3.4. Capsaicinoids and Capsiate Content

The resulting extracts were filtered through a 0.22 µm nylon syringe filter (Membrane Solution, Dallas, TX, USA) and 3 µL of the extract was injected into an ultra-high-performance liquid chromatography (UHPLC) equipment in order to identify the capsaicinoids and the capsiate. This equipment was coupled to a Quadrupole-Time-of-Flight Mass Spectrometer (Q-ToF-MS) (Synapt G2, Waters Corp., Milford, MA, USA) fitted with an Electrospray Ionization Source (ESI). The spectra were acquired in the *m*/*z* = 100–600 and the compounds that were identified with their corresponding *m*/*z* ratios were as follows: nordihydrocapsaicin (n-DHC) 294, capsaicin (C) 306, dihydrocapsaicin (DHC) 308, homocapsaicin (h-C) 320, homodihydrocapsaicin (h-DHC) 322, and capsiate (CTE) 307. Masslynx software version 4.1 was used to control the equipment and for the acquisition, integration and analysis of the data. The operating conditions, the parameters of the equipment components, and the variables used were the same as in one of our previously published works [39].

The chromatographic separations and quantifications were performed on an ACQUITY UPLC H-Class system (Waters Corp., Milford, MA, USA). This equipment comprised four main parts: A Quaternary Pump System, an Auto Sampler with temperature control, a Photodiode Array Detector (PDA), and a Waters ACQUITY UPLC BEH rp-C18 column (100 × 2.1 mm, 1.7 µm particle size). Empower 3 software (Waters Corp., Milford, MA, USA) was employed to control the equipment and for data analysis. The identified compounds were quantified based on their calibration curves. All the instrumental parameters, as well as the analytical characteristics, including the calibration curves followed the method previously developed by our team [39]. The chromatographic analyses were performed in duplicate, and the results were expressed in milligrams of compound per gram of fresh pepper (FW).

### 3.5. Statistical Analysis

The results were estimated as the mean ± standard deviation (SD) values of two replicates of each maturation stage. The resulting means were compared by Tukey’s test to determine if the differences were significant for *p* < 0.05. All the data analyses were performed by means of Statgraphic Centurion Version XVII (Statgraphics Technologies, Inc., The Plains, VA, USA).

## 4. Conclusions

The current work has demonstrated that pepper fruits from the varieties known as ‘Filius Green’ and ‘Filius Blue’ may undergo significant variations with regards to their content in the bioactive compounds that are responsible for pepper pungency, i.e., capsaicinoids and capsinoids. Such differences should be attributed to genotype intrinsic characteristics, since both varieties were grown in a greenhouse under the same controlled conditions. The ‘Filius’ varieties can be used for decorative and aesthetic purposes in foods and dishes due to their colorful fruits, for nutritional purposes and as a medicinal supplement because of their high content in capsaicinoids and capsinoids.

In this study, it has been seen that pepper fruit color experiences a number of changes over its ripening process, which have been analyzed in relation to the content of capsaicinoids and capsinoids throughout the fruit maturation. The possibility of eating peppers with similar hot sensations and different colors, as well as peppers of the same color with different pungency, which can be attractive to the consumer, has been observed. On the one hand, their optimal harvesting time should be determined by the fruit color in relation to the desired gastronomic application, which would be purple or green in the early stages of fruit development and red in the final stages. On the other hand, and considering their attractive biological activities exhibited by the compounds of interest that have been studied herein, when harvested for medical purposes, peppers should be collected at the moment when its bioactive content is the greatest or the pungent taste is the required. While both varieties analyzed follow the same trend for capsinoids accumulation and reach their maximum value on the 34th dpa, the content curve for capsaicinoids differs between the two varieties, so that the highest concentration levels were reached either on the 41st or the 69th dpa. C was the major capsaicinoid found in both varieties, followed by DHC, n-DHC, h-C, and h-DHC, and their content percentages hardly varied over the fruit maturation process. Based on the standardized values of each capsaicinoid, different patterns have been registered for h-C in comparison to the rest of the capsaicinoids.

## Figures and Tables

**Table 1:** Color of ‘Filius’ pepper fruits at the different stages of their development (dpa: days post-anthesis).

Start of Fruit Development	dpa	Color of the Fruits	
		‘Filius Green’	‘Filius Blue’
17/09	13	Green	Brown/Purple
10/09	20	Green	Purple
03/09	27	Green	Purple
27/08	34	Green/Red	Purple/Red
20/08	41	Green/Red	Red
13/08	48	Red	Red
06/08	55	Red	Red
30/07	62	Red	Red
23/07	69	Red	Red
16/07	76	Over-ripeness	Over-ripeness

**Table 2:** Standardized values (%) of individual capsaicinoids during the fruit development of the two varieties analyzed.

(dpa)		13	20	27	34	41	48	55	62	69	76
**‘Filius Green’**	**n-DHC**	12.49	25.66	33.77	76.11	73.54	98.58	89.03	94.43	100.00	63.49
	**C**	6.96	11.27	17.78	69.48	58.12	85.76	73.82	88.54	100.00	65.75
	**DHC**	10.33	13.43	19.58	65.83	55.50	93.78	83.52	94.20	100.00	61.76
	**h-C**	12.62	22.15	45.57	74.49	68.94	100.00	77.35	85.56	87.04	66.92
	**h-DHC**	20.83	24.34	30.84	62.77	61.29	95.43	77.14	84.74	100.00	69.98
**‘Filius Blue’**	**n-DHC**	37.41	47.41	69.05	92.43	100.00	77.56	82.14	83.57	74.46	62.53
	**C**	36.81	45.91	58.07	88.85	100.00	60.13	57.19	59.84	55.56	49.59
	**DHC**	19.12	25.77	44.81	82.24	100.00	42.80	39.81	46.94	39.47	33.25
	**h-C**	32.64	36.08	67.50	98.33	88.68	86.53	100.00	83.88	73.67	86.17
	**h-DHC**	13.40	24.78	45.22	75.21	100.00	70.37	66.47	72.12	62.67	59.93

## Supplementary Materials

The following are available online at https://www.mdpi.com/2223-7747/9/9/1222/s1, Figure S1: Representative chromatograms for the two varieties of pepper studied ((**A**) ‘Filius Blue’; (**B**) ‘Filius Green’) obtained by UHPLC-PDA (280 nm) at (1) 13 dpa; (2) 34 dpa; (3) 55 dpa; (4) 76 dpa. The identified compounds were: (1) Nordihydrocapsaicin (n-DHC); (2) Capsaicin (C); (3) Dihydrocapsaicin (DHC); (4) Homo-capsaicin (h-C); (5) Homo-dihydrocapsaicin (h-DHC); (6) Capsiate (CTE).
