# Supplementary material for: Content of Capsaicinoids and Capsiate in “Filius” Pepper Varieties as Affected by Ripening
Source: Plants (Basel). 2020 Sep 17;9(9):1222. doi: 10.3390/plants9091222 (PMC7569991; doi:10.3390/plants9091222)
Supplement: Supplementary file 1 [file plants-09-01222-s001.pdf]

SUPPLEMENTARY MATERIAL

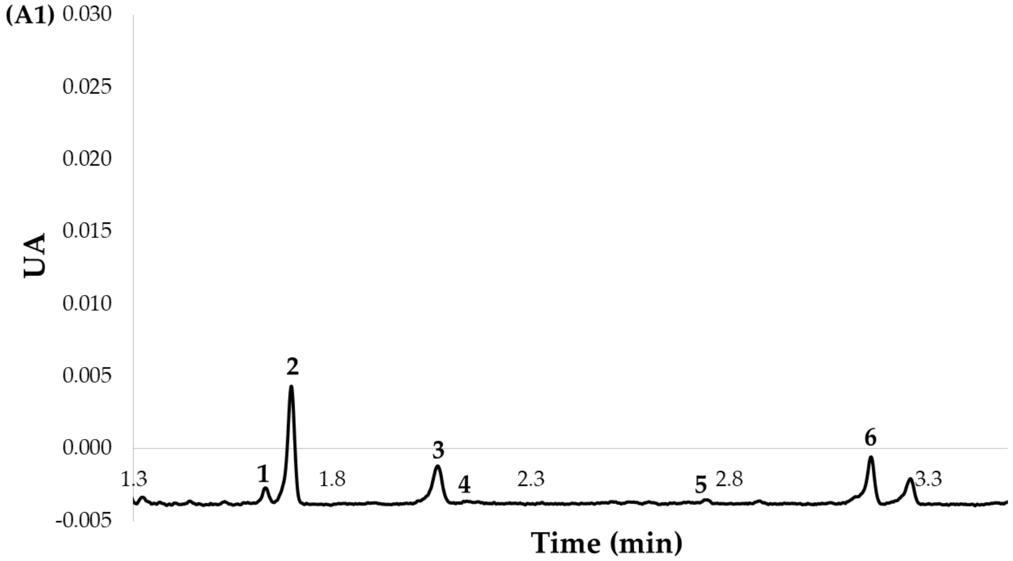

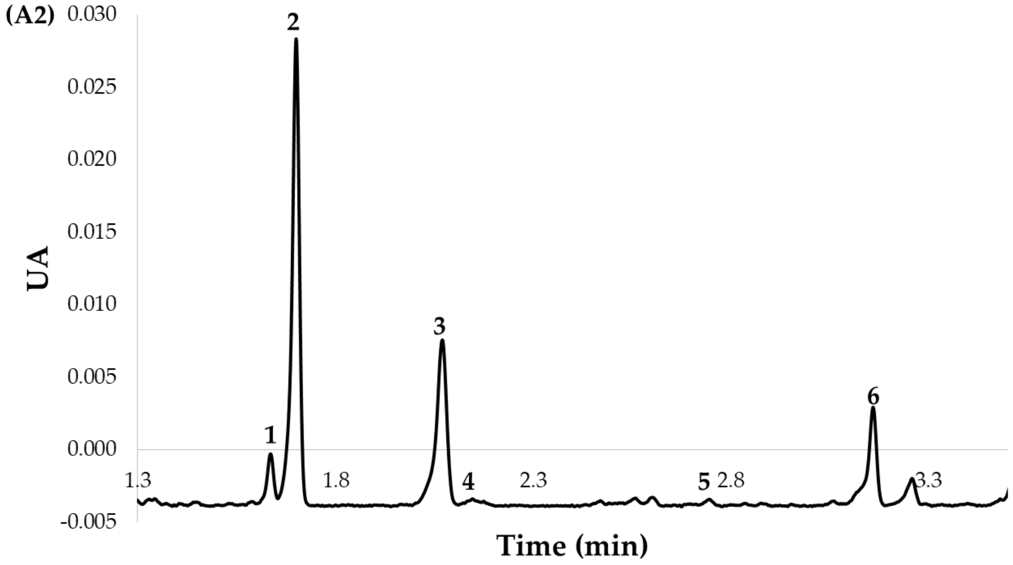

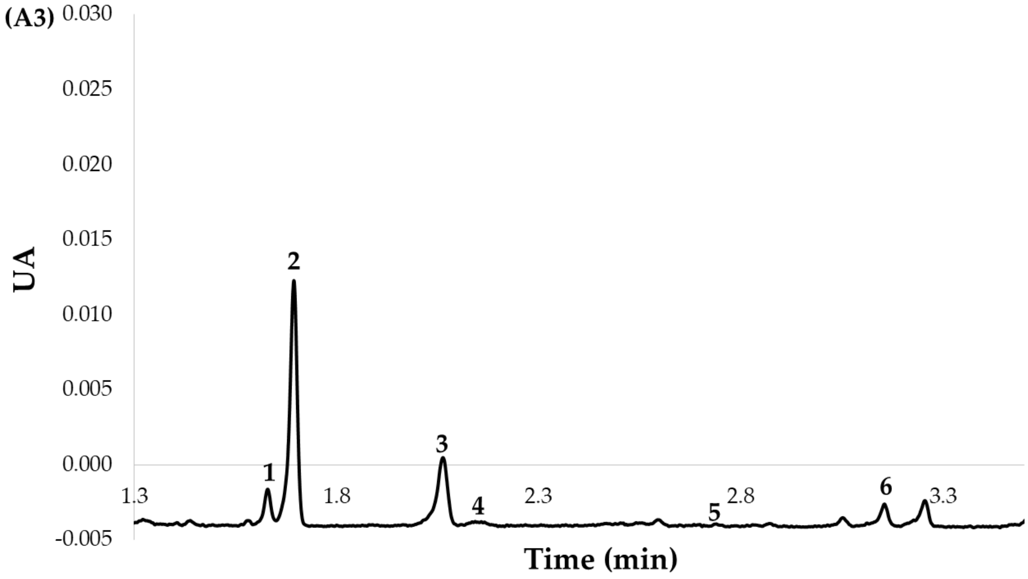

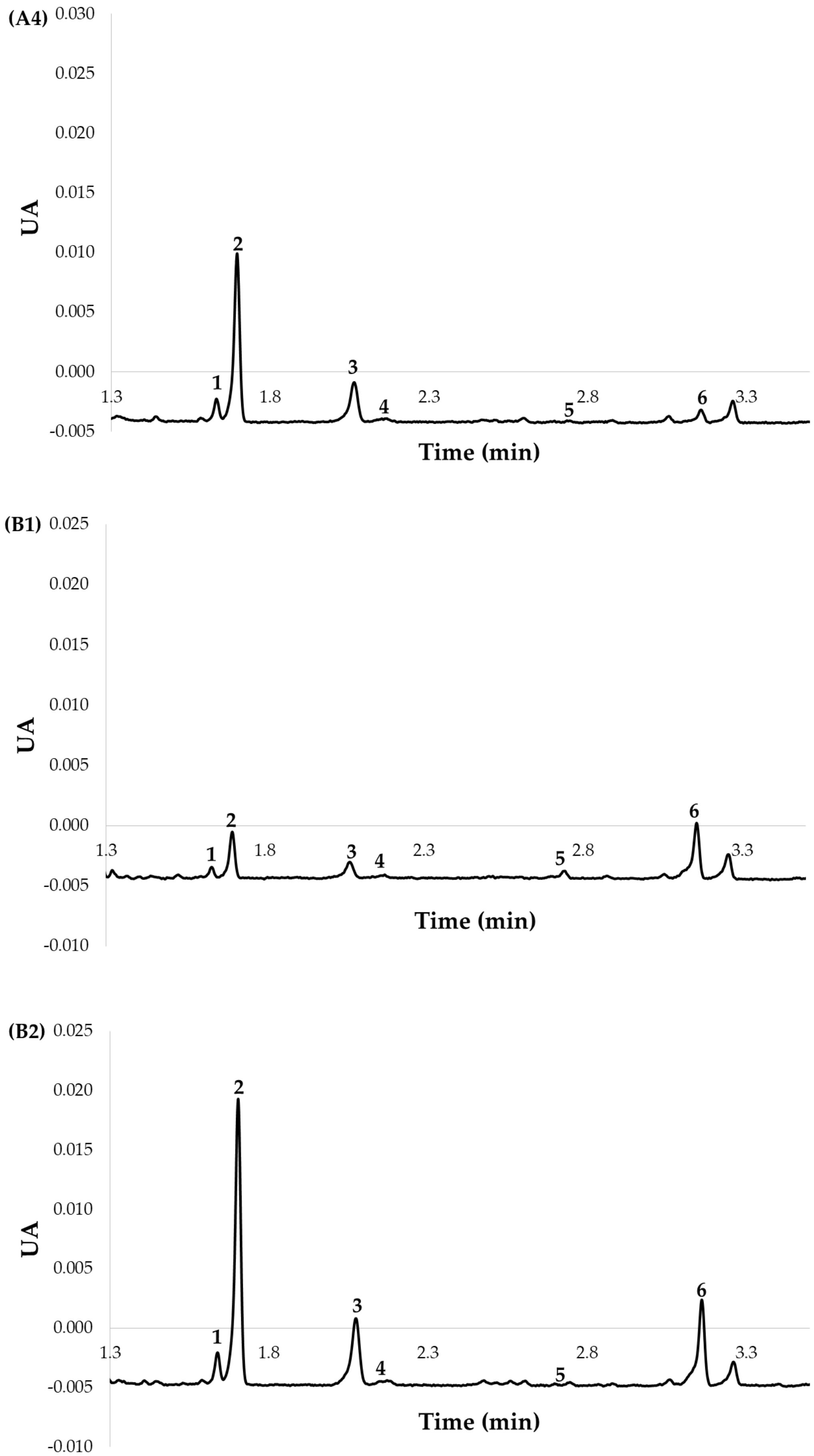
(A4)
0.030
0.025
0.020
0.015
0.010
0.005
0.000
-0.005
UA
1.3
1.8
2.3
2.8
3.3
1
2
3
4
5
6
Time (min)
(B1)
0.025
0.020
0.015
0.010
0.005
0.000
-0.005
-0.010
UA
1.3
1.8
2.3
2.8
3.3
1
2
3
4
5
6
Time (min)
(B2)
0.025
0.020
0.015
0.010
0.005
0.000
-0.005
-0.010
UA
1.3
1.8
2.3
2.8
3.3
1
2
3
4
5
6
Time (min)

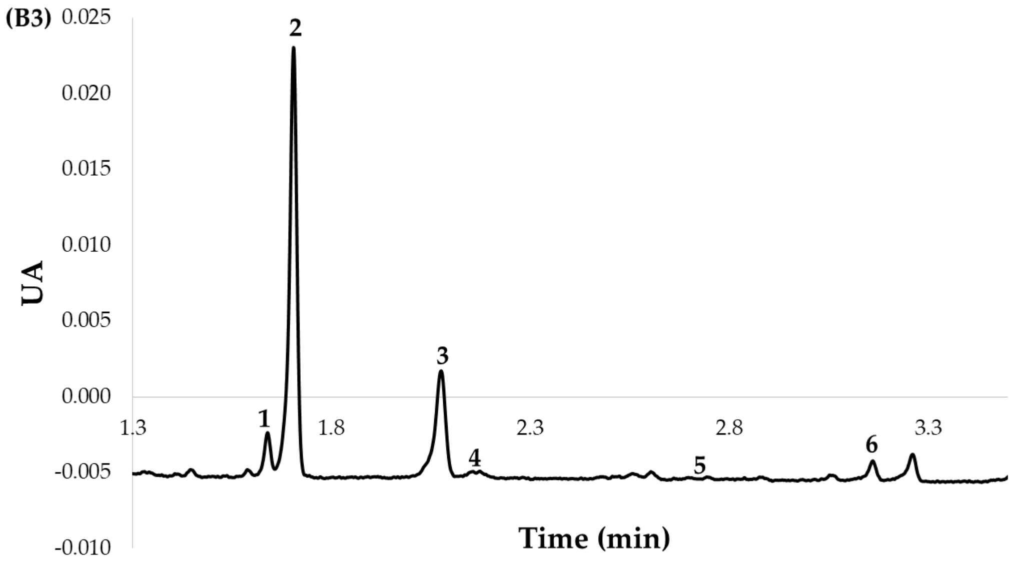

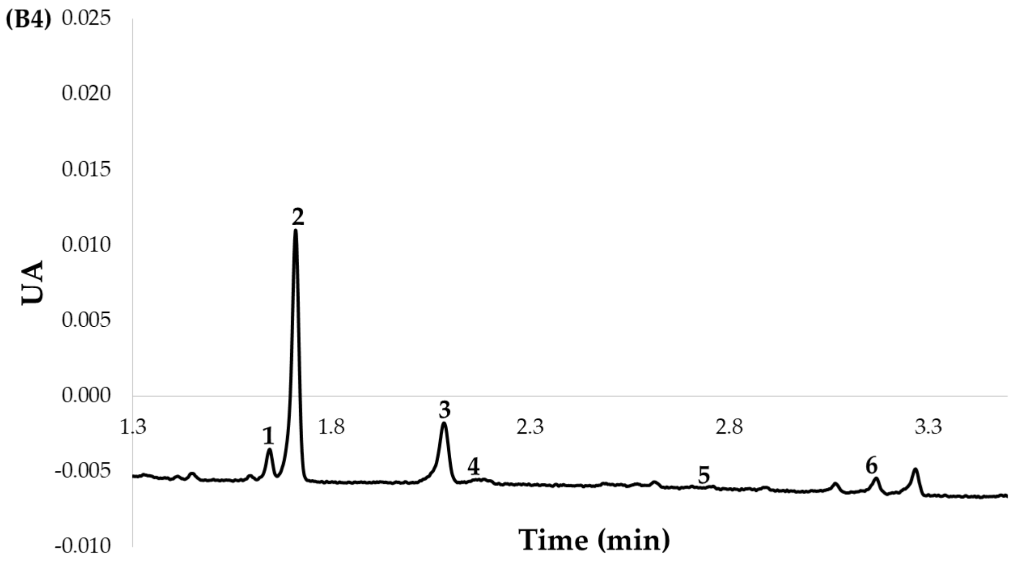

Figure S1. Representative chromatograms for the two varieties of pepper studied ((A) 'Filius Blue'; (B) 'Filius Green') obtained by UHPLC-PDA (280 nm) at (1) 13 dpa; (2) 34 dpa; (3) 55 dpa; (4) 76 dpa. The identified compounds were: (1) Nor-dihydrocapsaicin (n-DHC); (2) Capsaicin (C); (3) Dihydrocapsaicin (DHC); (4) Homo-capsaicin (h-C); (5) Homo-dihydrocapsaicin (h-DHC); (6) Capsiate (CTE).
